# Numerical Study on Mechanical Behavior and Electromechanical Properties of Solder-Jointed REBCO-Coated Conductors

**DOI:** 10.3390/ma17112517

**Published:** 2024-05-23

**Authors:** Tianfa Liao, Wenyuan Wang, Zhiming Chen, Mingzhi Guan

**Affiliations:** 1 College of Electronic Information and Electrical Engineering, Huizhou University, Huizhou 516007, China; ltf@hzu.edu.cn (T.L.); wwy@hzu.edu.cn (W.W.); chenzhiming@hzu.edu.cn (Z.C.); 2 Institute of Modern Physics, Chinese Academy of Sciences, Lanzhou 730000, China; 3 Advanced Energy Science and Technology Guangdong Laboratory, Huizhou 516000, China; 4 Key Laboratory of Mechanics on Western Disaster and Environment, Ministry of Education, College of Civil Engineering and Mechanics, Lanzhou University, Lanzhou 730000, China

**Keywords:** REBCO CC tapes, soldering lap joint, mechanical behavior, electromechanical properties, finite element model

## Abstract

As the second-generation high-temperature superconducting conductors, rare earth–barium–copper–oxide (REBCO) coated conductor (CC) tapes have good potential as high-field and high-energy superconductors. In superconducting applications, several joints are required for conjugating comparatively short REBCO CC tapes. Soldering lap joints are the simplest and most commonly applied REBCO CC joints. In addition to joint resistance, the mechanical behavior and electromechanical properties are also crucial for superconducting applications. In this paper, the electromechanical properties and mechanical behaviors of soldering lap joints at 77 K under a self-field were studied. The mechanical behavior was addressed by using a full three-dimensional multilayer elastic–plastic finite element model (FEM) with REBCO CC tape main layers and solder connecting layers. Then, the electromechanical properties were analyzed by using Gao’s strain-$I_{c}$ degradation general model on the basis of the FEM results. Both the mechanical behavior and electromechanical properties were verified by experimental results. The effects of soldering lap conditions including lap length, soldering thickness and lap style on the electromechanical properties and mechanical behaviors were discussed. The results indicate that shorter overlap lengths and a thinner solder can reduce the premature degradation of *I_c_* due to stress concentrations nearby the joint edges; moreover, the irreversible critical strain is significantly higher in the back-to-back joint approach compared to the widely used face-to-face joint approach.

## 1. Introduction

As second generation (2G) high-temperature superconducting (HTS) tapes, HTS rare earth–barium–copper–oxide (REBCO) coated conductor (CC) tapes exhibit high current capabilities and excellent mechanical properties when exposed to strong magnetic fields. REBCO CC tapes have great potential for high-field and high-energy applications, including fusion reactors [1,2,3], nuclear magnetic resonance [4,5,6,7], accelerator magnets [8,9], generators [10], magnetic energy storage [11], fault current limiters [12] and power cables [13].

In recent years, with the evolution of REBCO bulks [14,15], REBCO CC tapes have undergone a revolutionary development, e.g., by improving REBCO CC tape industrial production technology, manufacturers can construct single REBCO CC tapes longer than 1 km with especially uniform and high critical currents (*I_c_*) [16,17,18]. However, they still cannot meet the requirements of large applications including magnet coils and power cables which are several kilometers long [19,20]. Hence, fabrication of joints to connect REBCO CC tapes is a key component of the repair, fabrication, and development of superconducting devices.

Numerous techniques for main joints, including soldering joints [21], diffusion joints [22], melting diffusion joints [23], ultrasonic welding [24], and hybrid welding [25] were suggested and used for the fabrication of 2G HTS tapes for obtaining required lengths for coils and cables [26]. Soldering lap joints are the most commonly applied practical joint technique because of their fast and simple manufacturing procedures. In soldering joints, joint resistance is inevitable because of multiple resistive material layers, for example, the resistance of SuperPower SCS4050 REBCO CC tapes with pre-tinned solder joints is as high as ~1000 nΩ-cm^2^ [27]. Furthermore, mechanical characteristics vary for soldering joints and single tapes when applying deformation and stress [28]. Because REBCO CC tapes are very mechanically sensitive, excessive stress/strain can degrade their superconducting performance [29,30,31]. The study in [28] showed that the critical tensile strains of a single tape and a soldering joint were equal to 0.42% and 0.36%, respectively, indicating that mechanics-induced superconducting performance degradation is more pronounced in soldering joints due to the stress concentration. Hence, investigation of the electromechanical properties and mechanical behaviors of solder joints is necessary.

Several tests have been performed for the investigation of the electromechanical properties and mechanical behaviors of solder joints under various loading conditions [21,22,23,24,25,27,32,33,34,35,36,37]. Among them, axial tension experiments are commonly applied as a fundamental approach for the examination of solder joint performance [28,33,34,35,36,37]. It is shown that the performance of solder joints is significantly different from that of single tapes, both in terms of mechanical and electromechanical properties. Additionally, various simulations have been performed for the analysis of strain and stress distributions of joint components under mechanical loads [28,35,37,38,39]. Huang et al. [28] and Konstantopoulou et al. [37] used FEM to simulate the distribution of the stress in the spliced joint samples under axial loading; furthermore, Peng et al. [39,40] analyzed the interface cracking and stress distribution characteristics of joints under mechanical deformation based on a cohesive zone model. However, the available numerical studies can only provide the strain and stress distribution of each component in a solder joint, but the intrinsic mechanisms of the mechanical behavior and electromechanical properties of the joints have rarely been studied. Thus, it is extremely difficult to quantitatively predict and optimize the structural configuration of joints. This motivates us to carry out a systematic numerical and theoretical study on the electromechanical properties and mechanical behaviors of solder joints.

In the current study, using a full 3D multilayer elastic–plastic FEM with REBCO CC tape main layers, solder connecting layers were constructed to investigate soldering lap joint mechanical behavior, and electromechanical characteristics were further characterized by the longitudinal strain dependence of the *I_c_* phenomenological model based on FEM results. After the validation of mechanical behavior and the electromechanical properties, the effects of soldering lap conditions including soldering thickness, overlap length and lap style on electromechanical properties and mechanical behaviors were discussed. This study provides a method for effectively predicting and evaluating the electromechanical properties of REBCO soldering lap joints, and the related results can provide guidance for the optimal design of joint structures.

## 2. Models Description

### 2.1. 3D Elastoplastic FEM

**Figure 1:**
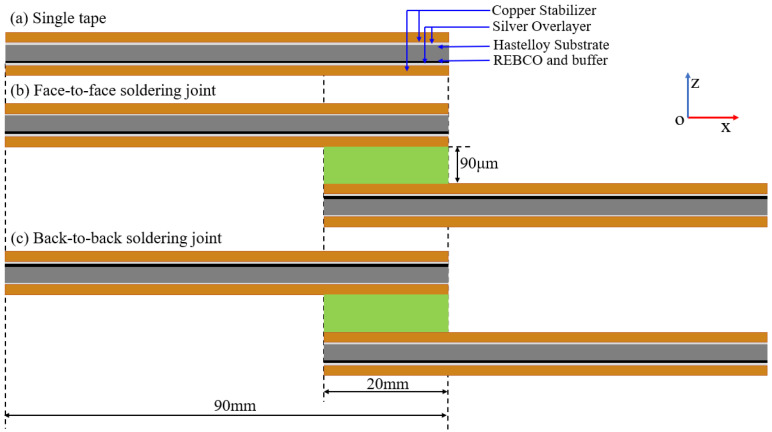
Schematic diagram of simulated structures.

Solder joint REBCO CC geometry is obtained using SuperPower^®^ SCS4050 [40]. The thickness and width of the CCs are 95 μm and 4 mm, respectively. The CC tape is a multilayer structure, including two layers of copper, two layers of silver, REBCO + buffer layers and Hastelloy substrate, as shown in Figure 1a, with thicknesses of 2 × 20 μm, 2 × 2 μm, 1.2 μm and 50 μm, respectively [28]. The Hastelloy substrate and the side of the REBCO layer in the REBCO CC tapes were named “back” and “face”, respectively. A face-to-face configuration (as shown in Figure 1b) is usually employed to reduce joint resistance as much as possible. The corresponding back-to-back joint approach (as shown in Figure 1c) with higher joint resistance was also comparatively analyzed in this study. According to the available literature [28,37], an overlap joint length of 20 mm and solder thickness of 90 μm were adopted. To evaluate the mechanical behaviors of REBCO CC joints and tapes under tensile loading, full 3D multilayer elastic–plastic FEMs were implemented using COMSOL Multiphysics. 

**Figure 2:**
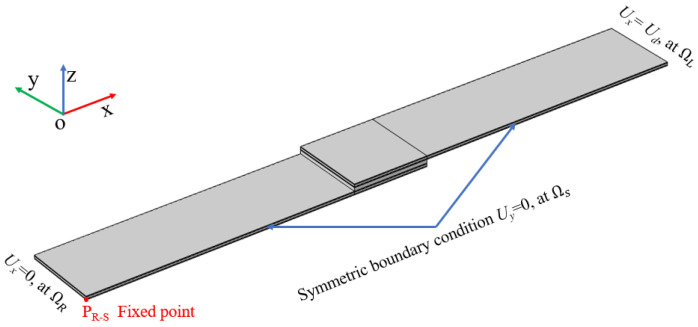
Schematic boundary conditions for FEM.

Considering the symmetry of the structure and to save the computational cost, a 1/2 symmetric model was adopted for the FEM, as shown in Figure 2, where Ω_S_ is the symmetric boundary. To achieve uniaxial tensile loading, the displacement in the tape length direction (x-axis direction) is constrained to be 0 at one end of the model (Ω_R_), and the uniaxial tensile displacement load is applied through the displacement boundary condition at the other end (Ω_L_). To limit rigid body displacement, a fixed constraint needs to be defined at one point (P_R-S_) of the intersection line of the boundary between Ω_R_ and Ω_S_.

For the finite element mesh, we used a structured hexahedral mesh containing 34,320 mesh nodes as well as 29,400 hexahedral mesh elements in the soldering lap-joint model. Because the area near the overlapping boundary of the joint is the most important area in this study, the element size is a gradient grid to make the mesh denser near the overlapping boundary of the joints. 

In the current research, it has been supposed that Hastelloy, copper, silver, REBCO and solder were isotropic, continuous, and homogeneous materials. A linear elastic model was applied for REBCO and a bilinear elastoplastic model was applied for Hastelloy, solder silver, and copper. The von-Mises yield criterion was adopted for defining the yield onset of plastic materials. All material properties at 77 K under a self-field applied in FEM are presented in Table 1 based on multiple sources [41,42,43,44]. Interfacial failure is not considered in this study; therefore, displacement continuum constraints are adopted for the different material interfaces.

### 2.2. Strain Dependence of $I_{c}$ Model

The HTS conductor tape mechanical load effect on $I_{c}$ can be divided into irreversible and reversible regions. In the reversible region, Ekin’s power law was applied for depicting reversible variations of $I_{c}$ under various mechanical loads [45,46,47,48,49]. In the irreversible region, the Weibull distribution function was applied to depict the $I_{c}$ irreversible degradation of BSCCO [50,51,52,53,54] and the REBCO CC tapes [42,43,50,55,56,57] under mechanical loads. 

Gao et al. developed a phenomenological $I_{c}$ model for the prediction of $I_{c}$ tensile strain dependence in 2G HTS tapes in both irreversible and reversible regions [42]. The model was modified for the prediction of the REBCO CC tape’s electromechanical properties under non-uniform combined deformations, including bending tension [56] and combined tension–torsion [43]. Gao’s model successfully predicts that irreversible strain can be increased by encapsulating the REBCO CC tapes, thereby improving the electromechanical performance of the REBCO CC tape [57]. A short discussion of Gao’s model for $I_{c}$ strain dependence is presented below.

Under mechanical load, critical current density ($j_{c}$) normalized with critical current density at zero strain ($j_{c0}$) is presented by the phenomenological model combining the Weibull distribution function and Ekin’s power-law formula [43]:

(1) $$\frac{j_{c}}{j_{c0}}=\frac{1}{1-a|\varepsilon_{\max}{|}^{b}}(1-a|\varepsilon_{L}-\varepsilon_{\max}{|}^{b})W(\varepsilon_{L})$$

where $\varepsilon_{\max}$ is longitudinal strain at maximum $j_{c}$, *a* and *b* are constants of Ekin’s power-law formula, and $\varepsilon_{L}$ is longitudinal strain. The Weibull distribution function *W*($\varepsilon_{L}$) depends on longitudinal strain, which is stated as:

(2) $$W\left(\varepsilon_{L}\right)=\left\{ \begin{array}{cc} \exp\left[-{\left(\frac{\varepsilon_{L}-\varepsilon_{irrc}}{\varepsilon_{0c}}\right)}^{m_{c}}\right] & \left(\varepsilon_{L}<\varepsilon_{irrc}\right)\\ 1 & \left(\varepsilon_{irrc}\leq \varepsilon_{L}\leq \varepsilon_{irrt}\right)\\ \exp\left[-{\left(\frac{\varepsilon_{L}-\varepsilon_{irrt}}{\varepsilon_{0t}}\right)}^{m_{t}}\right] & \left(\varepsilon_{L}>\varepsilon_{irrt}\right) \end{array}\right.$$

where ${mt\;\left(mc\right),\varepsilon }_{0t}$ ($\varepsilon_{0c}$), and $\varepsilon_{irrt}$ ($\varepsilon_{irrc}$), and are shape parameters, scale parameters, and irreversible strains of $I_{c}$ under tensile (compressive) load, respectively. The $I_{c}$ value could be obtained by the integration of $j_{c}$ across REBCO film cross-sections. Accordingly, $I_{c}$ normalized by the critical current ($I_{c0}$) under zero strain is stated as:

(3) $$\frac{I_{c}}{I_{c0}}=\frac{1}{S_{0}}\iint_{S_{0}}\frac{1}{1-a{\left|\varepsilon_{\max}\right|}^{b}}\left(1-a{\left|\varepsilon_{L}-\varepsilon_{\max}\right|}^{b}\right)W\left(\varepsilon_{L}\right)dxdz$$

where $S_{0}$ is REBCO film cross-sectional area under zero mechanical load.

In this study, Gao’s model is not only used to study the electromechanical behavior and validation analysis of a single REBCO CC tape but is also used to try to predict the electromechanical properties of a joint, so as to investigate the electromechanical degradation mechanism of a joint under mechanical loads and give quantitative optimization analyses.

## 3. Results and Discussions

### 3.1. Computational Validations

To ensure the accuracy of the FEM and to investigate the capability of Gao’s model in the prediction of strain dependence of *I_c_* in solder joints, validation evaluation of the electromechanical properties and mechanical behaviors of single REBCO CC tapes as well as solder joints were first carried out. Face-to-face solder joints are widely used because of their lower joint resistance. Therefore, all joints studied in this paper refer to face-to-face solder joints if not otherwise specified.

#### 3.1.1. Single Tape

The accuracy or otherwise of the calculation results of the electromechanical properties and mechanical behaviors of a single REBCO CC tape directly affects the reliability of the relevant results of the solder joints. Here, we first carry out experimental validation of the calculated results for the stress–strain as well as strain-*I_c_* characteristics of a single REBCO CC tape.

- Mechanical behavior

**Figure 3:**
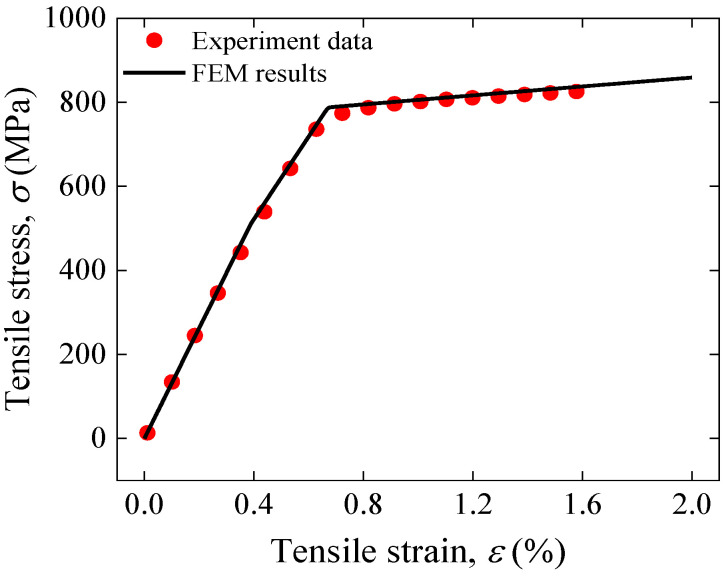
Comparison of tensile stress versus applied tensile strain between single REBCO CC tape simulated results and experimental data [43].

Figure 3 illustrates the stress–strain relationship simulation results under tension for REBCO CC tape at 77 K under self-field. Here, tensile stress σ and tensile strain ε are calculated as engineering strain and engineering stress [43] as:

(4) $$\varepsilon =\frac{l-l_{0}}{l_{0}},$$

(5) $$\sigma =\frac{F}{A_{0}},$$

where $A_{0}$ is the conductor in the original cross-section area, F is the applied tension load applied in the simulations, *l* is the conductor’s final simulated length, and $l_{0}$ is the conductor’s original length. The results show that REBCO CC tape stress–strain curves are consistent with experimental data [43], indicating that the establishment of composite CC tape FEM and the material parameter selection are reasonable.

- Electromechanical properties

**Figure 4:**
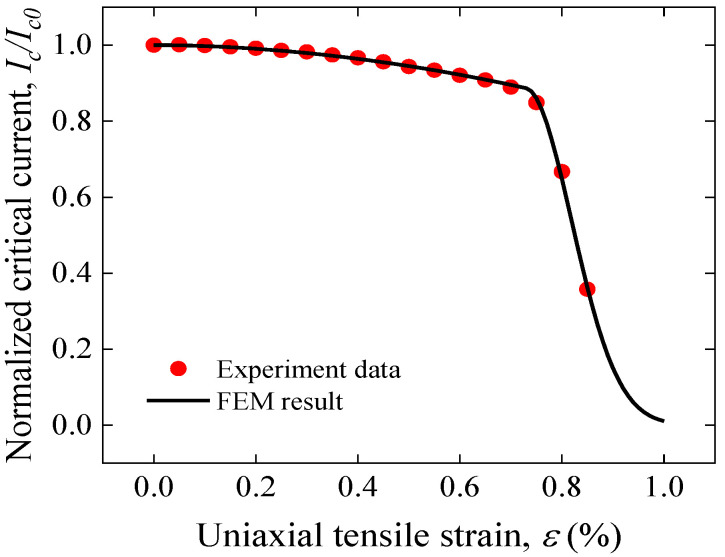
Comparison of Gao’s experimental data and model predictions for tensile strain dependence of normalized $I_{c}$ of a single REBCO CC tape [43].

Using Gao’s model described by Equations (1)–(3), the dependence of $I_{c}$ on tensile strain can be studied. Because this is a case of uniaxial tensile deformation, none of the compression-related model parameters are considered. Thus, the parameters in Equation (1) used in [43] were directly adopted as *a* = 1300, *b* = 1.9, $\varepsilon_{0}=0.13\%$, $\varepsilon_{max}=$ 0 and $\varepsilon_{irr}=0.73\%$. Based on the longitudinal strain of the superconducting layer obtained by FEM with Gao’s model and the corresponding model parameters, the critical current characteristics under uniaxial strain can be predicted. Figure 4 compares the experimental data and model results for the uniaxial tensile strain dependence of normalized $I_{c}$. Model predictions agreed well with experimental results for the irreversible and reversible degradation stages. 

#### 3.1.2. Solder Joint

- Mechanical Behavior

**Figure 5:**
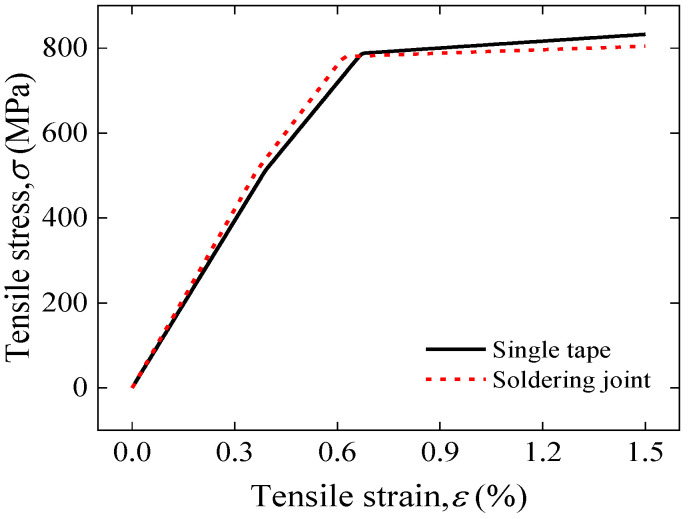
Comparison of applied tensile strain and tensile stress between single tape and solder joint.

**Figure 6:**
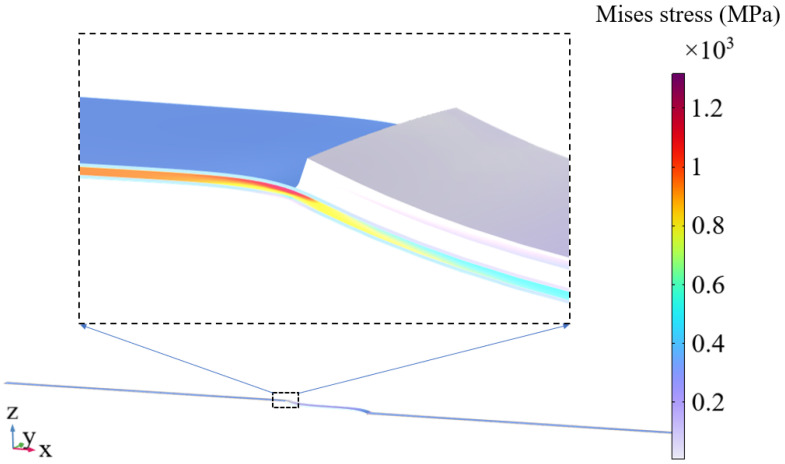
Deformation diagram (displacements magnified 10×) and stress distribution for applied strain equal to 0.5%.

Figure 5 compares applied tensile strain with tensile stress between single tape and 2 cm solder lap-jointed REBCO CC tapes. It can be seen that the two stress–strain curves are basically the same, except that the yield strain and yield stress of the joint are slightly lower than that of the single strip. The results are consistent with the experimental phenomenon [28]. As shown in Figure 6, this is due to the torsional and bending deformation at the edge of the joint overlap region under tensile loading resulting in a concentration of stress at the edge of this region, causing localized premature yield failure.

- Electromechanical properties

**Figure 7:**
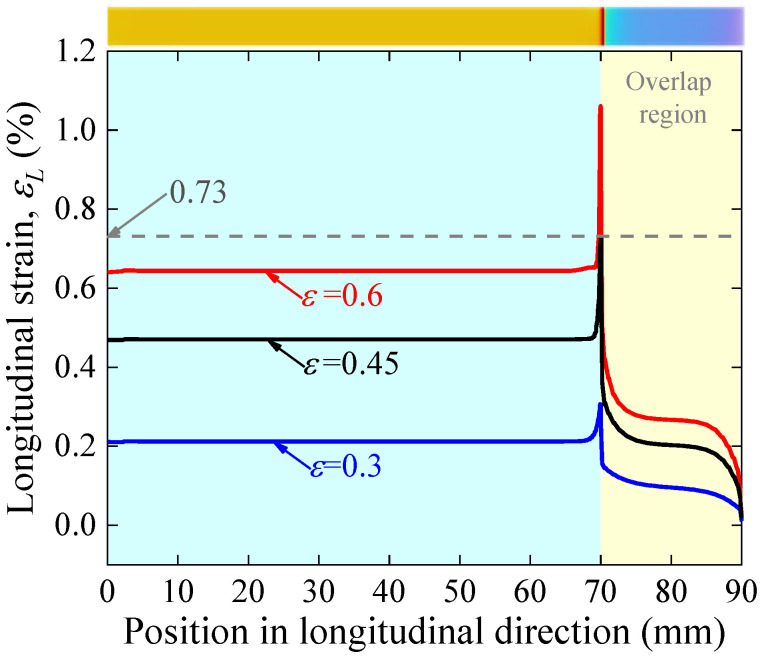
Longitudinal strain distribution in REBCO superconducting layer for applied strain equal to 0.3%, 0.45% and 0.5%. The inset is a cloud view of the longitudinal strain distribution in the superconducting layer.

The inhomogeneous stress further leads to the inhomogeneous distribution of strain along the length of the joint. Figure 7 shows the distribution characteristics of the longitudinal strain of the REBCO superconducting layer on one joint side along the longitudinal direction for different applied strains. Uniform strain distribution is observed in the part away from the joint, whereas there is a sudden increase at the overlap edge and a gradual decrease in the overlap region. This indicates that the mechanical failure as well as the electromechanical degradation starts at the edges of the overlap joint. It is noteworthy that the superconducting layer’s maximum longitudinal strain reaches 0.73% (the irreversible strain) when the applied axial strain is 0.45%; it can be deduced that the *I_c_* of the joint will irreversibly degrade at an applied strain of 0.45%.

**Figure 8:**
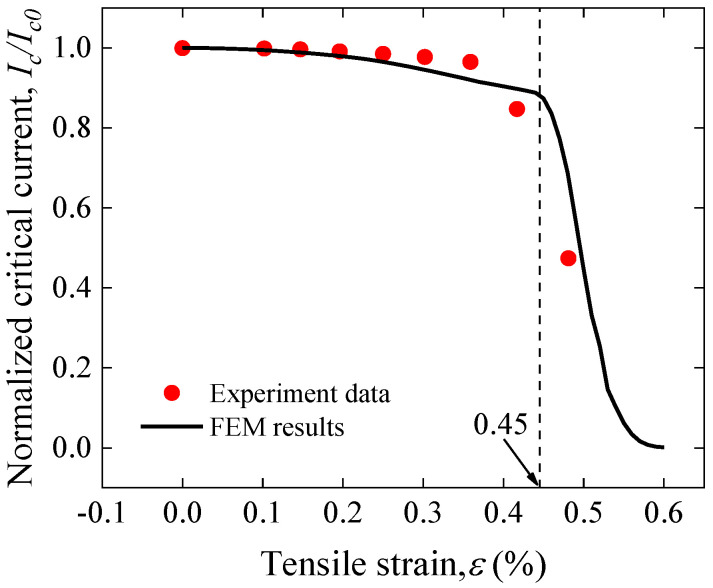
Comparison of experimental data and Gao’s model predictions for the tensile strain dependence of normalized $I_{c}$ of a face-to-face solder joint [43].

It is well known that REBCO CC tape’s current-carrying capacity is obtained from the REBCO film’s weakest cross-section; therefore, joint current-carrying capacity is obtained from the cross-section of the REBCO superconducting film with the largest longitudinal strain, i.e., the position of the strain mutation at the edge of the overlap region, as shown in Figure 7. Therefore, the normalized *I_c_* for this REBCO section was calculated based on Gao’s model and the model parameters used in the single tape. The results are shown in Figure 8 that when the applied strain reaches 0.45%, a sharp degradation in the *I_c_* occurs, which is consistent with the previous strain analysis. The model predictions agree well with the experimental results, which further proves that Gao’s model is also capable of accurately predicting the solder joint electromechanical properties and reveals that the mechanism of the degradation of the solder joint electromechanical properties lies in the premature degradation of the current-carrying capability due to the concentration of stress at the edges of the joints.

### 3.2. Discussion

After verifying FEM accuracy in the solder joint mechanical calculation and the effectiveness of Gao’s strain-$I_{c}$ model in predicting the electromechanical properties, we can safely carry out a quantitative analysis and discussion of the structural configuration of solder joints as well as the lap styles. Specifically, we discuss the effects of overlap length, solder thickness, and lap style on the mechanical behavior and electromechanical properties, respectively, and the structural configurations of the solder joints used in each case are shown in Table 2.

#### 3.2.1. Effect of Overlap Length

**Figure 9:**
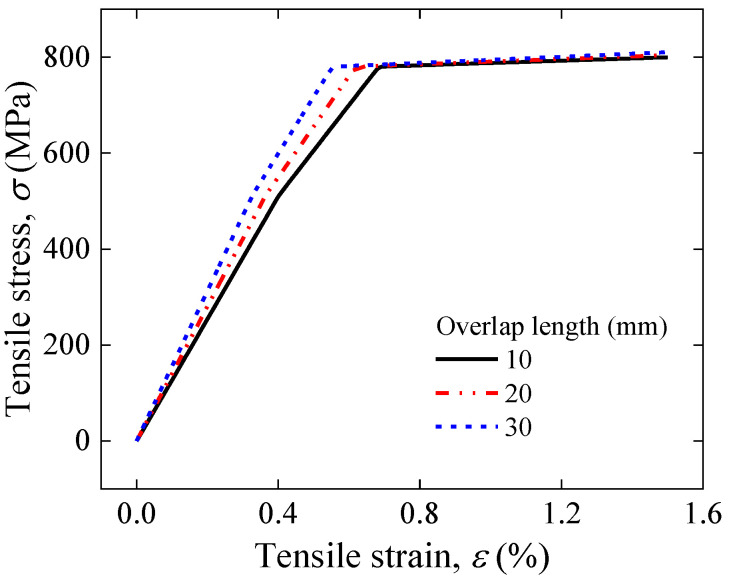
Comparison of applied tensile strain and tensile stress for an overlap length of 10–30 mm of face-to-face solder joints.

**Figure 10:**
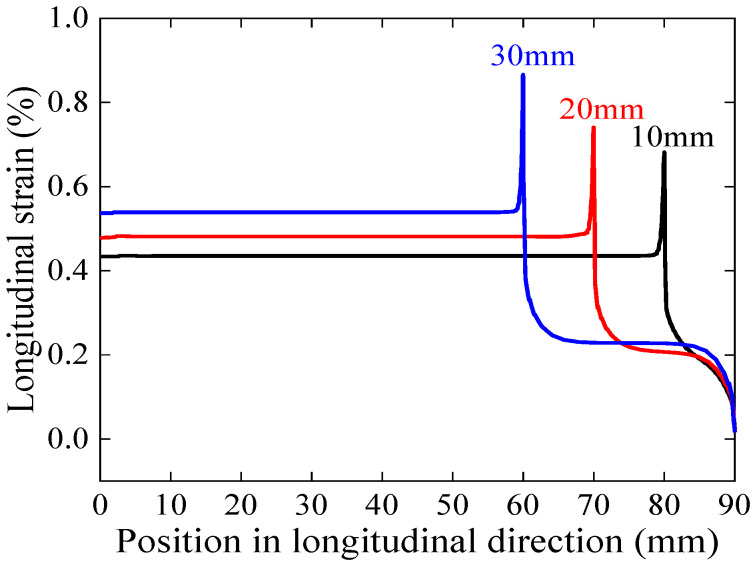
Longitudinal strain distribution in REBCO superconducting layer for an overlap length of 10–30 mm of face-to-face solder joints under applied strain of 0.45%.

**Figure 11:**
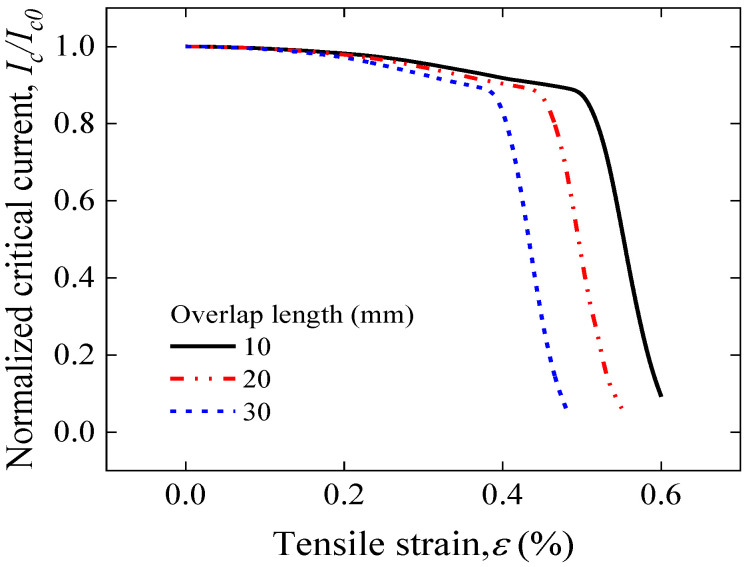
Comparison of tensile strain dependence of normalized $I_{c}$ predicted by Gao’s model for an overlap length of 10–30 mm of face-to-face solder joints.

Figure 9 illustrates the stress–strain curve simulation results for the overlap length of 10–30 mm of face-to-face solder joints. The stress–strain curves corresponding to different overlap lengths have almost the same yield stress, but the yield strain decreases with increasing overlap length. This implies that excessive joint lengths will result in a decrease in the safe strain range. The maximum longitudinal strain of the joint increases with increasing overlap length for the same applied strain shown in Figure 10, further implying that longer overlaps will lead to premature degradation of the superconducting current-carrying properties under mechanical loads. The results for the electromechanical properties predicted by Gao’s model shown in Figure 11 also validate this idea; as the length of the overlap increases, the irreversible strain decreases, and the degradation of *I_c_* is more severe under external loading.

#### 3.2.2. Effect of Solder Thickness

**Figure 12:**
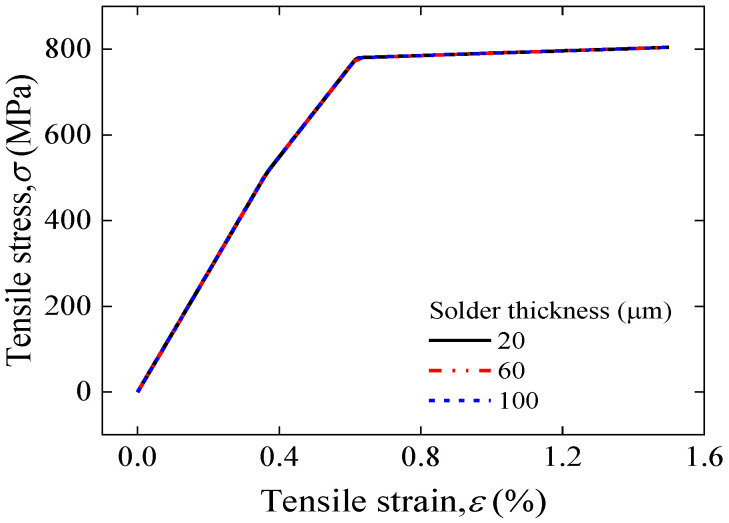
Comparison of applied tensile strain and tensile stress for a solder thickness of 20–100 μm of face-to-face solder joints.

**Figure 13:**
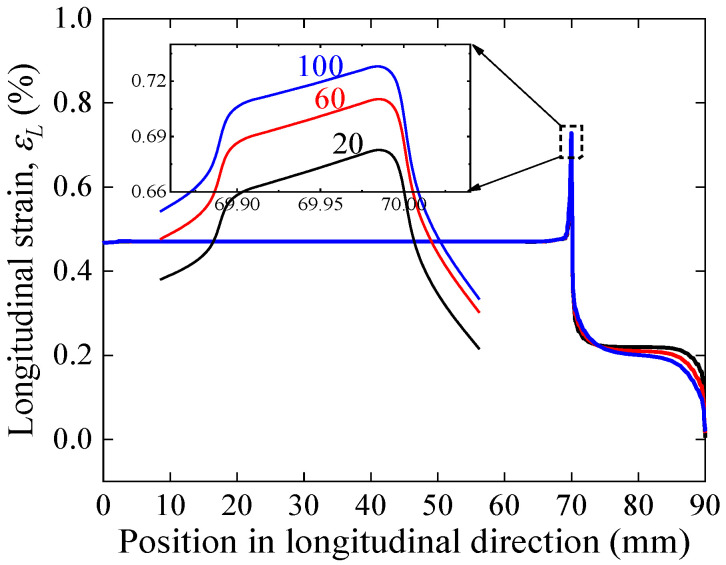
Longitudinal strain distribution in the REBCO superconducting layer for a solder thickness of 20–100 μm of face-to-face solder joints under applied strain of 0.45%.

**Figure 14:**
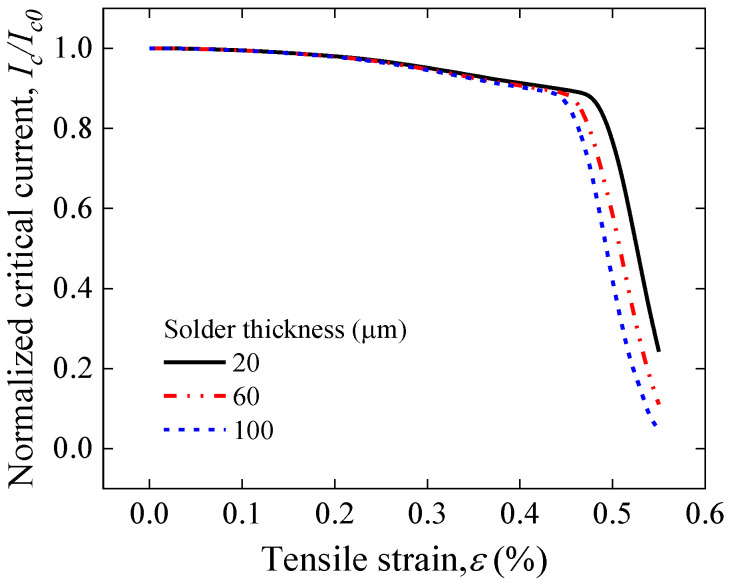
Comparison of tensile strain dependence of normalized $I_{c}$ predicted by Gao’s model for a solder thickness of 20–100 μm of face-to-face solder joints.

Figure 12 illustrates the stress–strain curve simulation results for a solder thickness of 20–100 μm of face-to-face solder joints. The results show that the curves almost overlap completely, indicating that the thickness of the solder within a reasonable range has no effect on the overall mechanical load carrying capacity of the joints. Figure 13 further displays the longitudinal strain distribution in the REBCO superconducting layer for a solder thickness of 20–100 μm under the same applied strain. The results indicate that the maximum longitudinal strain of the joint increases with increasing solder thickness, further implying that joints with greater thicknesses of solder will lead to the premature degradation of superconducting current-carrying properties under mechanical loads. The results of the electromechanical properties predicted by Gao’s model shown in Figure 14 also validate this idea; as the thickness of the solder increases, the irreversible strain decreases, and the degradation of *I_c_* is more severe under applied loads.

This discussion indicates that an appropriate reduction in thickness of solder has almost no effect on mechanical behavior, but it can increase the irreversible strain of *I_c_* degradation, thereby enhancing the electromechanical properties of the solder joint.

#### 3.2.3. Effect of Lap Style

The face-to-face lap-joint structure can minimize joint resistance and is a commonly used solder joint method. However, in this type of joint, the lap joint will undergo torsional and bending deformation under tensile loads, resulting in tensile bending in the REBCO superconducting layer, leading to a steep increase in the REBCO superconducting layer’s local longitudinal strain and thus causes early degradation of *I_c_*. Ignoring the high resistance in the joints, a back-to-back joint approach might provide a higher safe strain range. The back-to-back joint approach is comparatively analyzed in this section. 

**Figure 15:**
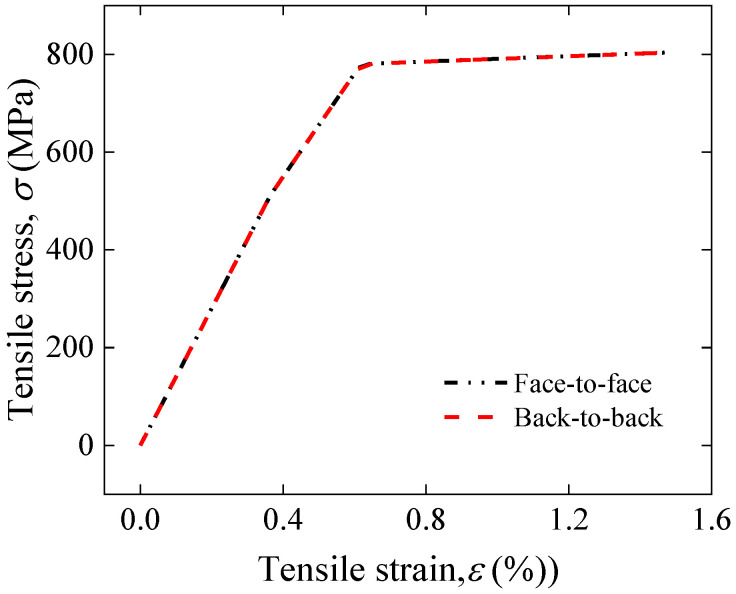
Comparison of applied tensile strain and tensile stress between face-to-face and back-to-back joints.

Figure 15 shows a comparison of the simulation results of stress–strain curves between face-to-face and back-to-back joints. The results show that the two curves overlap completely. This is due to the fact that only the joint method was changed and not the components and proportions of the joint. This indicates that the use of the back-to-back joint method has no effect on the overall mechanical load-carrying capacity of the joints.

**Figure 16:**
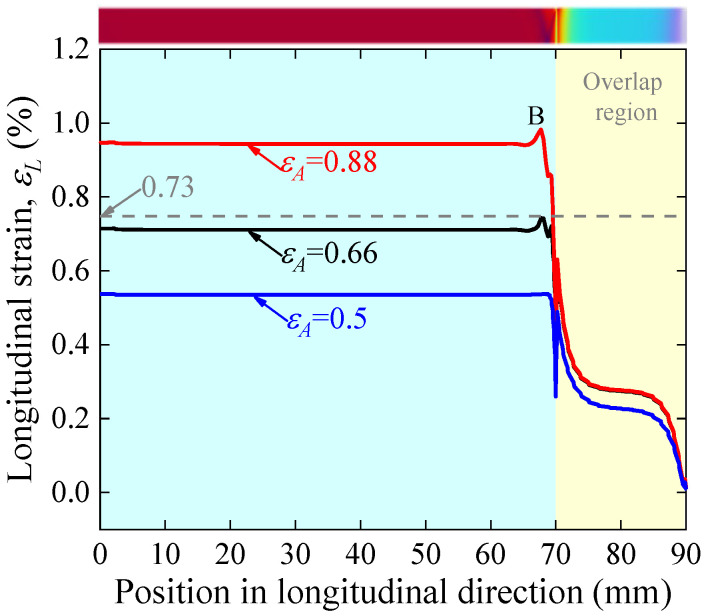
Longitudinal strain distribution in the REBCO superconducting layer of a back-to-back joint for applied strain equal to 0.5%, 0.66% and 0.88%. The inset is a cloud view of the longitudinal strain distribution in the superconducting layer.

To determine the most dangerous section, the distribution characteristics of the REBCO superconducting layer’s longitudinal strain on one joint side along the longitudinal direction is shown in Figure 16. As in the face-to-face scenario, the strain distribution is uniform in the part away from the overlap region, and there is a gradual decrease in strain in the overlap region. Unlike the face-to-face case, there is a sharp drop rather than an increase at the overlap edge. This is due to the compressive bending deformation in the REBCO superconducting layer near the edge of the overlap when the back-to-back joint is subjected to tensile loads. Another feature that differs significantly from the face-to-face scenario is a small rise in strain near the overlap edge (point B). REBCO CC tape’s current-carrying capacity is obtained from the REBCO superconducting layer’s cross-section corresponding to point B. For 0.66% applied axial strain, the maximum longitudinal strain of the REBCO superconducting layer reaches 0.73%, indicating that the Ic of the back-to-back joint will irreversibly degrade at an applied strain of 0.66%.

**Figure 17:**
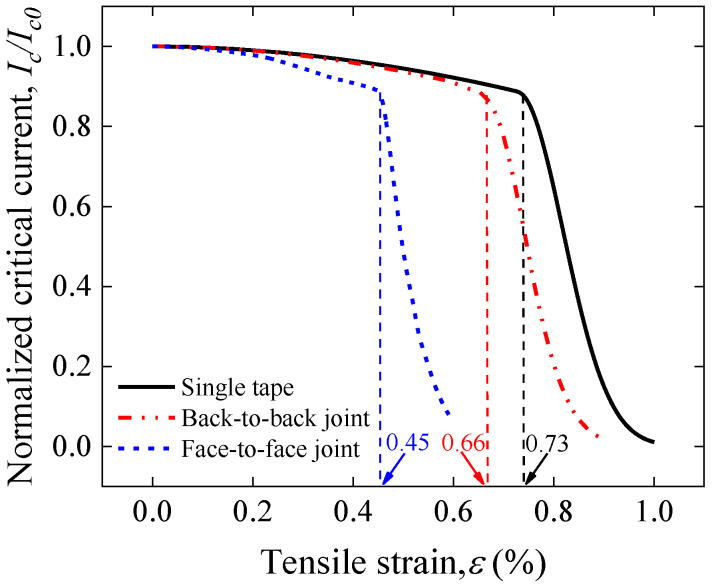
Comparison of tensile strain dependence of normalized $I_{c}$ predicted by Gao’s model for single REBCO CC tape, back-to-back joint and face-to-face joint.

The normalized $I_{c}$ of the back-to-back joint calculated based on Gao’s model and the model parameters used in the single tape is shown in Figure 17. For comparative analysis, the normalized $I_{c}$ of the single tape and face-to-face joint are also shown. When the applied strain reaches 0.66%, a sharp degradation of the *I_c_* occurs for the back-to-back joint, which is consistent with the previous strain analysis. Although the *I_c_* degradation irreversible strain of the back-to-back joints is smaller than that of the single tape, it is significantly higher (47% increase) compared to that of the face-to-face joints. This indicates that the back-to-back jointing method can effectively reduce the degradation of superconducting current-carrying properties due to stress concentration at the joints. This result also provides new insights into solder joints, where back-to-back joints should also be a candidate because they provide a higher stress/strain range than face-to-face joints, provided the joint resistance meets the requirements of the application.

## 4. Conclusions

In this study, using a full 3D multilayer elastic–plastic solder lap-joint FEM with main layers of REBCO CC tapes, solder connecting layers were constructed to investigate the mechanical behavior of soldering lap joints, and the electromechanical properties were further characterized by Gao’s strain-$I_{c}$ model with longitudinal strain calculated by FEM. Both electromechanical properties and mechanical behaviors were experimentally verified. The results confirm that Gao’s model can be used to accurately predict solder joints’ electromechanical properties and reveal that the mechanism for the electromechanical degradation of solder joints is the premature degradation of current-carrying capacity due to stress concentration at the overlap zone edge. Furthermore, the effects of soldering lap conditions including overlap length, soldering thickness and lap style on electromechanical properties were discussed. The discussion shows that the degradation of the *I_c_* of soldering lap joints is more severe under external loading with a longer overlap and thicker solder. Thus, shorter lap length with a thinner solder is preferred as this can reduce the premature degradation of the $I_{c}$ of a solder joint. Additionally, the irreversible critical strain is significantly higher in the back-to-back joint approach compared to the widely adopted face-to-face joint approach. Thus, back-to-back joints can be a candidate for solder joints as well.

## Figures and Tables

**Table 1:** Material characteristics of silver, REBCO, Hastelloy solder, and copper [41,42,43,44].

	Tangent Modulus $E_{t}$ (GPa)	Yield Strength $\sigma_{s}$ (MPa)	Poisson’s Ratio ν	Young’s Modulus *E* (GPa)
Silver	1	17	0.37	76
Copper	5	340	0.34	85
Hastelloy	5	1200	0.307	178
Solder	0.1	60	0.3	30
REBCO	——	——	0.3	157

**Table 2:** Structural configuration of solder joints for discussions.

	Overlap Length (mm)	Solder Thickness (μm)	Lap Style
Case 1	10–30	90	face-to-face
Case 2	20	60–100	face-to-face
Case 3	20	90	face-to-face and back-to back

## Data Availability

The original data supporting the research are not publicly available but a portion of the data that is not confidential is available on request from the corresponding author.
